# Sex chromosome abnormalities and psychiatric diseases

**DOI:** 10.18632/oncotarget.13962

**Published:** 2016-12-15

**Authors:** Xinzhu Zhang, Jian Yang, Yuhong Li, Xin Ma, Rena Li

**Affiliations:** ^1^ Beijing Institute for Brain Disorders, Capital Medical University, Beijing, China; ^2^ Beijing Key Laboratory of Mental Disorders, Beijing Anding Hospital, Beijing, China; ^3^ Center for Hormone Advanced Science and Education, Roskamp Institute, Sarasota, FL, USA

**Keywords:** schizophrenia, autism, ADHD, depression, gender

## Abstract

Excesses of sex chromosome abnormalities in patients with psychiatric diseases have recently been observed. It remains unclear whether sex chromosome abnormalities are related to sex differences in some psychiatric diseases. While studies showed evidence of susceptibility loci over many sex chromosomal regions related to various mental diseases, others demonstrated that the sex chromosome aneuploidies may be the key to exploring the pathogenesis of psychiatric disease. In this review, we will outline the current evidence on the interaction of sex chromosome abnormalities with schizophrenia, autism, ADHD and mood disorders.

## INTRODUCTION

**Figure 1:**
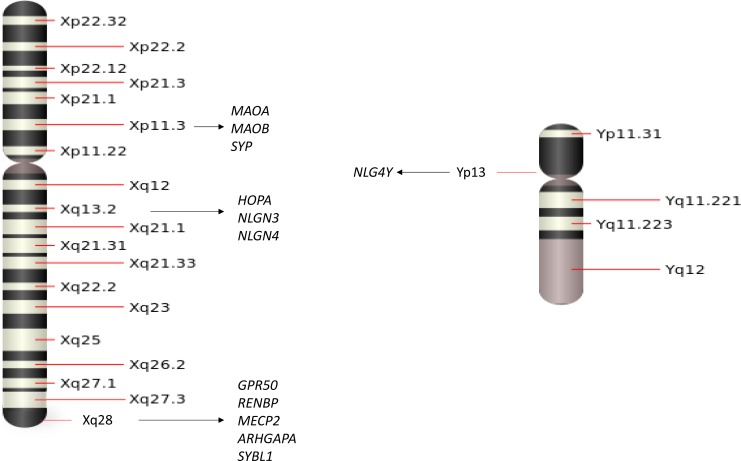
A schematic of the sex chromosomes with several adjacent genes that have been associated with psychiatric diseases

Sex chromosome abnormalities are the most common chromosomal abnormalities in humans [1]. Sex chromosome aneuploidies can influence neurodevelopment and often result in more difficulties in inhibition, mental flexibility, sustained attention, working memory, verbal skills and executive function impairment [2], while some of these symptoms partly overlap with psychosis. Studies have demonstrated that genes in the sex chromosome may influence psychiatric disease by altering the basic differentiation process of the neurons [3], encoding proteins [4], synaptic transmission [5] and so on. For example, X-linked genes have specific impacts on the development of the amygdala and its connections with cortical centers involved in social-cognition processing as shown in Figure 1 [6], while structural abnormalities of the amygdala in individuals have been proven to increase risk for schizophrenia [7]. Therefore, the study of sex chromosomes in psychiatric diseases may provide a new angle to understand the sex differences in the pathogeneses of psychiatric diseases. In this review, we gathered discoveries in psychiatric research and discussed the relationship between sex chromosomes and psychosis in order to provide some useful insights on sex-specific genetic mechanisms for psychiatric disease.

## GENES IN SEX CHROMOSOMES AND PSYCHIATRIC DISEASES

An important category of genetic linkage with sex chromosomes is that the genes on X or Y chromosomes not only determine male and female traits but also carry many sex related characteristics. While men are the only ones who inherit Y chromosomes and Y-linked traits, both men and women can get X-linked genes since both inherit X chromosomes. There are 1,098 known human X-linked genes and only about 26 known genes located in the Y chromosome. Some X or Y chromosome genes have been identified as candidate genes for psychiatric disease, such as schizophrenia (SCZ), major depression disorder (MDD), bipolar disorder (BPD), attention-deficit hyperactivity disorder (ADHD), autism spectrum disorder (ASD). In this session, we will highlight some genes located on X or Y chromosomes and summarize their linkage with these psychiatric disorders. As shown in Figure 1, we will review each of the genes and their connection to specific psychiatric diseases.

### The human opposite paired-containing gene (*HOPA*) in SCZ

*HOPA* is an X-chromosome gene that codes for a critical family of proteins regulating transcription via the nuclear receptor. Accumulating studies of HOPA exonic polymorphisms in SCZ patients suggested that *HOPA^12bp^*, an exonic polymorphism, plays critical roles in neuronal growth and differentiation and is associated with increased risk for SCZ, concluding that the *HOPA*^12bp^ allele is a risk factor for SCZ in subjects of European ancestry [8–11]. Furthermore, a study reported that *HOPA*^12bp^ allele is associated with the negative symptoms of SCZ by comparing the positive and negative symptoms in 43 male *HOPA*^12bp^ SCZ patients and 137 *HOPA* ^wild^ controls [11]. However, a study of 99 SCZ patients (56 males, 43 females) in the Chinese population reported no association of *HOPA* with SCZ [12]. Although the association between *HOPA* polymorphisms and SCZ in different populations remains unclear, another study of 367 SCZ patients and 178 BPD patients from Bulgaria and UK showed little to no correlation between *HOPA*^12bp^ and SCZ and BPD [13]. Controversial findings hypothesized that mutations may not occur directly on *HOPA*^12bp^, but in other genes in the Xq13 region, especially those that have neurobiological function. For example, the human neuroligin-3 gene is the closest to the *HOPA* gene. Neuroglin gene’s putative promoter region overlaps the last exon of the *HOPA* gene located less than 900 bp from the position of the *HOPA*^12bp^ [14].

### G-protein-coupled receptor 50 (GPR50) in mood disorders and SCZ

*GPR50* is located in X chromosome Xq28 and has been studied as a candidate gene for BPD, MDD and SCZ. A good example is a case-control study of subjects with BPD (*n* = 264), MDD (*n* = 226) and SCZ (*n* = 263) with ethnically matched controls (*n* = 562). The study discovered an association between an insertion/deletion polymorphism in exon 2 of *GPR50* and BPD and MDD, while other single-nucleotide polymorphisms (SNPs) within *GPR50* showed associations between MDD and SCZ. However, all of the associations were found only in females, suggesting a sex-specific risk of *GPR50* for BPD, MDD and SCZ[15]. A similar sex-specific risk of *GPR50* in other psychiatric disorders has been reported by other independent studies. For instance, by genotyping 400 men and 610 women who had depression for more than 12 years, studies found a female-specific association between *GPR50* variants and later-life depression (specific for depression combined with anxiety), and female patients showed more inclination for currently having depression or anxiety than male patients [4]. Furthermore, a study of 106 seasonal affective disorder patients (80 females and 26 males) found that female patients with BPD (36%) or MDD (60%) are related to *GPR50* variants. The sex-specific association between the variant of *GPR50* and mood disorders in females suggest that *GPR50* may be a female-specific risk for mood disorder [16]. A recent large cohort study of *GPR50* polymorphisms in 1010 elderly men and women showed that women with heterozygous for rs13440581 showed an increased risk of depression and the risk increased when depression was combined with anxiety in women homozygous for rs2072621. Another *GPR50* polymorphism rs561077 was also associated with an increased risk of incident depression specifically. However, no significant associations were observed in men [4]. Together, studies of *GPR50* support that *GPR50* may contribute to a female-specific risk for SCZ, BPD, and MDD.

### Neuroligin 3 gene (*NLGN3*), neuroligin 4X gene (*NLGN4X*) in ASD

*NLGN3* and *NLGN4X* are the X-linked genes located in Xq13.1 and Xp22.31, respectively. Studies found an association between *NLGN3*, *NLGN4X* and ASD. By screening for *NLGN3 and NLGN4X* mutations in subjects with ASD or Asperger syndrome (a type of ASD with higher cognitive ability and normal language), one study of 140 males and 18 females identified two affected brothers; one has ASD and another has Asperger syndrome in one Swedish family. The two affected brothers shared the same frameshift mutation in *NLGN4X* with their mother while no mutations were found in other family members. In addition, a similar genetic phenomenon of *NLGN3* was found in another Swedish family in two brothers with ASD and Asperger syndrome, respectively. There is a C to T transition (changing arginine residue into cysteine) in *NLGN3* found in the two affected brothers, while the same mutation was also found in their mother, but not in other family members [17]. Furthermore, studies screened the full length of the coding regions of the *NLGN3* and *NLGN4X* genes in ASD patients and their parents, including nine nucleotides on *NLGN3* and *NLGN4X* gene to be associated ASD as indicated in Table 1. One study of 40 Greek autism patients and their parents found the c. - 705 A > G nucleotide change located in the *NLGN4X*, exon 1 of variant 1 in the father of a young girl who has ASD and the mother of a young boy who suffers ASD. In addition, the same study also found a 4-year-old boy with severe ASD inherited c. - 206G > C, is located in *NLGN4X* exon 2 from his healthy mother [18]. However, an independent study of 107 autism patients (102 males, 5 females) found no genetic variants for both X-linked genes *NLGN3* and *NLGN4X* in ASD on a high functioning level by screening four polymorphisms and one novel synonymous variant [19]. A most recent study reported that in addition to point mutations were identified in *NLGN4X* and *NLGN3* in patients with ASD, an elevated *NLGN4X* phosphorylation is also interplayed with the genetic mutation into the synaptic dysfunction of ASDs [20]. Together, data suggested that mutations on *NLGN3* and *NLGN4X* might increase the risk of autism while polymorphisms of different nucleotides in autism still need further investigation.

**Table 1:** List of X- or Y- genes about the psychosis disorder

Gene	Diagnosis	Subject	Comments	Year
*HOPA* (Xq13)	SCZ	Female SCZ (n=50), mothers of male SCZ (n=50)	Disease related variants: Prom 1, Exon 6b, Exon 15, Exon 23b, Exon 28, Exon 43	2003[8]
	SCZ	569 SCZ (378 males, 191 females)	11 male patients and 11 female patients have the *HOPA^12bp^*.	2007[9]
	SCZ	172 SCZ (128 males, 45 females); controls (1035 males, 471 females)	HOPA mutation rates are 4% male SCZ, 6% female SCZ, 1% male controls and 1.2% female controls.	2001[10]
	SCZ	180 male SCZ (43 *HOPA^12bp^*, 137 *HOPA ^wild^*)	HOPA genotypes are related with bizarre behavior, not with positive symptoms.	2004[11]
	SCZ	99 SCZ (56 males, 43 females)	No association in *HOPA* mutations and SCZ.	2003[12]
	SCZ BPD	367 SCZ (265 males, 102 females) and 368 controls (258 males and 110 females); 178 BPD (81 males and 97 females) and 188 controls (84 males and 104 females)	The risk of *HOPA^12bp^* were found higher in the SCZ and BPD than normal controls.	2003[13]
*GPR50* (Xq28)	MDD	Elderly MDD with 12 years’ depression incident (400 males, 610 females)	rs13440581, rs2072621, rs561077 are only significantly associated with females, not in males.	2015[4]
	SAD	106 SAD (80 females and 26 males)	rs2072621 SNP is significantly associated with SAD in females, but not in males.	2012[16]
	BPD SCZ MDD	264 BPD (121 males, 143 females), 263 SCZ (187 males, 76 females), 226 MDD (90 males, 136 females)	*GPR50^wt/D502–505^* polymorphism is both significantly associated with BPD and MDD in females.	2005[15]
*NLGN3 (*Xq13.1)*, NLGN4X* (Xp22.31)	ASD	40 ASD (36 males, 4 females) and 150 controls (89 males 61 females)	One SNP in *NLGN3* rs17857401; eight SNPs in NLGN4X: rs111953947, rs72413786, rs2290487, rs2290488, rs7049300, rs3747333, rs3747334, c.1597A>G (p.K378R).	2013[18]
	ASD	158 ASD (140 males and 18 females)	p.R451C in NLGN3 (Xq13 locus, OMIM 300336) and c.1186insT in *NLGN4X* (Xp22.3 locus, OMIM 300427).	2003[17]
	ASD	107 ASD (102 males, 5 females)	SNPs in NGLN4X: rs2290488, rs7049300, rs3747333, rs3747334, and one novel synonymous variant (A558).	2008[19]
*MAOA* (Xp11.3 )	SCZ	346 SCZ	No allelic association between *MAOA* and aggressive behavior measured by total OAS or OAS4.	2004[25]
	ADHD	1462 ADHD (1216 males and 246 females) and 807 controls (507 males, 300 females)	SNP in MAOA rs5905859 and in SYP rs5906754 are highly associated with ADHD compared to controls.	2014[3]
	ADHD	456 ADHA children (379 males, 77 females) and 108 normal controls (77 males, 31 females)	SNPs in MAOA: rs3788862, rs5905859, rs3027400, rs2239448, rs1137070.	2015[21]
	MDD	146 MDD (44 males, 102 females) and 101 controls (33 males and 68 females)	Higher activity of the MAOA gene promoter alleles is associated with MDD in females.	2000[22]
*MAOB* (Xp11.3)	ASD SCZ	142 ASD (122 males, 20 females); 143 SCZ (95 males, 48 females)	*MAOB* gene various p. R448X is associated with SCZ, not to ASD.	2011[24]
	SCZ	90 family subjects with two or more members with SCZ	the PAH 232 bp and the GABRB3 191 bp allele in *MAOB* are significantly association with the delusions factor and the hallucinations factor detected by QPDTPHASE, respectively.	2009[78]
	ADHD	150 ADHD probands (126 males, 24 females), 150 controls (125 males, 25 females)	Two of 7 functional variants were found to be polymorphic (rs2283728 ‘C’, rs3027440 ‘T’) alleles and associated with ADHD probands.	2016[23]
*MECP*2 (Xq28)	SCZ	498 SCZ and 2071 controls for GWAS; 1027 SCZ, 1001 controls, 71 affected offspring and 173 unaffected family members for follow up study	GWAS show 10 top candidate genes and 3 of the 10 genes were located within the Xq28 region (RENBP, ARHGAP4, MECP2). In follow up study, 4 SNPs were found in SCZ: rs2269372 in RENBP, rs2269368 in ARHGAP4, rs2734647 and rs2239464 in MECP2.	2014[27]
	ASD	69 females clinically diagnosed with ASD	One ASD carries a heterozygous 41-bp deletion at nucleotides 1157-1197 of MECP2, and another ASD carries a single nucleotide sequence change 880 C3T.	2003[28]
	ASD	59 ASD (42 males and 17 females)	No mutation in *MECP2* gene coding region in ASD.	2001[29]
*SYBL1* (Xq28)	BPD	110 BPD and 119 controls	four single nucleotide polymorphisms were detected in BPD and only frequency of C allele showed a trend toward to BPD in males.	2000[30]
	BPD	164 BPD (67 males, 97 females), and 267 controls (165 males, 102 females)	The C allele showed be more frequent in males with BPD than in respective controls.	2002[31]
*NLGN4Y*(Yq 11.22)	ASD	26 boys (XYY) and 11 males control (XY)	*NLGN4Y* correlated with XYY in the autistic mannerisms symptom t-scores.	2015[32]

Notable: SCZ= Schizophrenia BPD= Bipolar Disorder SAD=Seasonal Affective Disorder MDD= Major Depressive Disorder ADHD= Attention Deficit Hyperactivity Disorder ASD= Autism Spectrum Disorder

### Monoamine oxidase A or B (*MAOA* or *MAOB*) in ADHD, ASD, SCZ and depression

*MAOA* is a candidate gene located in Xp11.3 for various psychiatric disorders. For example, studies showed that the *MAOA* is associated with ADHD by Pedigree-based generalized multifactor dimensionality reduction (PGMDR) on 1462 ADHD children and 807 unaffected controls, [3] while another study found that *MAOA* variants may be associated with sustained attention deficit by genotyping DNA from ADHD children (*n* = 242) which evaluated the attention deficit by a digit cancellation test [21]. Interestingly enough, *MAOA* is also related to depression in female patients. Studies of *MAOA* genotypes (type 1 with at least one short allele 3, type 2 with only the long alleles 3.5, 4 or 5) in depressive patients and controls found that females with major depression had significantly higher frequencies of *MAOA* type 2 than that in female healthy controls, while no differences were found in *MAOA* type 1 between depression patients and controls regardless of genders [22]. In addition to *MAOA*, *MAOB* is also association with ADHD. A new study of 150 subjects with ADHD (126 males, 25 females) found higher frequencies of seven functional variants in *MAOB* (Table 1) in ADHD patients compared to controls (125 males and 25 females) [23]. *MAOB* is also thought to be one of the best candidate genes for ASD and SCZ by sequencing 113 candidate genes from 142 ASD patients and 143 SCZ patients [24]. However, the associations between *MAOA* or *MAOB* and SCZ have been challenged by several independent studies. A study analyzed 346 SCZ patients by the Overt Aggression Scale (OAS) found no support of the two genes as risk factors for positive symptoms such as aggressive behavior [25]. Another study found that *MAOB* is related with the etiology of psychotic diseases, but not specifically for SCZ while the authors failed to prove the association between *MAOB* and five psychotic disorder features, such as delusions, hallucinations, mania, depression, and negative symptoms [26].

### Methyl CpG binding protein 2 (*MECP2*) in SCZ and ASD

*MECP2*, a gene located on Xq 28 was identified in SCZ patients by a genome-wide association study (GWAS) on 498 SCZ patients and 2015 controls [27]. While the association between *MECP2* and ASD is still not clear, a recent study identified mutations in *MECP2* in two of the 69 female ASD patients, suggesting female-specific association between *MECP2* and ASD [28]. However, other study found no mutations or polymorphisms in the *MECP2* gene among 59 autism patients (42 males and 19 females) [29]. Therefore, whether *MECP2* is a x-chromosome related risk gene for autism remains further confirmation.

### Synaptobrevin-like 1 (SYBL1) in BPD

*SYBL1* is located on Xq28 maps to the Xq pseudoautosomal region and is a candidate gene for BPD. A study of 110 BPD patients (53 males and 57 females) and 119 control subjects (65 males and 54 females) by single-strand conformation polymorphism(SSCP) analysis and DNA sequencing found an increase in the frequency of *SYBL1* in males but not in female BPD patients [30]. As shown in Table 1, the sex-specific risk of *SYBL1* for BPD has been also supported by other studies. For instance, a study observed a significantly increased frequency of genotypes homozygous for the C allele, a novel polymorphism found on *SYBL1*, in males with BPD in comparison with male controls [31].

Moreover, several other X-linked genes were also observed as potentially relevant variants in ASD and SCZ (for example *IL1RAPL1*, *OPHN1*, *TM4SF2/TSPAN7*, or *MECP2*)^5^ as listed in Table 1.

### Neuroligin 4 Y (NLGN4Y) in ASD

Compared to the X chromosome, the Y chromosome is relatively understudied. *NLGN4Y* is located in Yp13 and is related to autism. A study of 26 boys with an extra copy of the Y chromosome (XYY) diagnosed with autism scales had the expression two-fold higher level of *NLGN4Y* than that in normal controls [32]. There are other Y-linked genes like *SPR and STS* which are expressed in the brain and influence sex-related brain structure [33], but no evidence of a relationship between these genes and psychiatric disease. Due to the limited psychiatric disease-related proteins were encoded by the Y-chromosome, the potential impact of Y-linked genes on psychiatric disease has been less investigated [34].

## SEX CHROMOSOME ABNORMALITIES IN PSYCHIATRIC DISEASES

Sex chromosome abnormalities include chromosome inactivity impairment, X chromosome escaping, additional copies of sex chromosomes (aneuploidies) and more. We will discuss the sex chromosome abnormalities in several major psychiatric disorders in this section.

### Schizophrenia (SCZ)

The loss or gain function of X chromosome in SCZ has been reported. For example, one study looked at mosaic X chromosome aneuploidies in 21 SCZ patients who had first-degree relatives with psychosis conditions and their healthy relatives, and found one SCZ patient who had X chromosome aneuploidy with 46XX/47XXX/48XXXX while her healthy mother carried 46XX/45X [35]. This data suggested that X chromosome aneuploidies might contribute more risk for familial SCZ. The high frequency of X aneuploidy among patients with SCZ has also been reported by other studies using structured diagnostic criteria and karyotypic laboratory analyses. In one meta analysis paper, using 34910 consecutive newborns as healthy controls, studies found that extra copies of X chromosomes, such as the frequency of 47XXY and 47XXX have a 4-6 times greater risk in SCZ patients compared to the sex chromosome aneuploidies in newborn controls [36]. The same paper also found a higher rate of XXX genotypes in female patients with SCZ. Interestingly, an individual with a missing X chromosome copy, such as XO was also reported in female SCZ patients (1 XO out of 11 SCZ patients) [37]. Furthermore, studies also reported that female SCZ patients carry XO karyotype (11/6483) three times more frequent than that in the general female population (9/17,038) [36, 38], which is consistent with some case reports showing XO karyotype in female SCZ patients [39, 40]. However, there are different results in terms of sex chromosome aneuploidies reported by studies with large clinical sample size. For instance, study found no association between the aneuploidies of X or Y chromosomes and risk of SCZ in 33512 persons who registered in The Danish Cytogenetic Central Register (DCCR) [37]. Among the 33512 individuals, there are 1122 cases with sex chromosome abnormally, including 18 (1.6%) SCZ patients (13 with 47XXY and 5 with 47XYY). The frequency of SCZ in 47XXY (72%) is higher than the risk of SCZ in general population(1%) [41]. Since it is known that sex chromosome aneuploidy increases with age in peripheral lymphocyte cultures [42], a study of longitudinal X chromosome aneuploidy can be done in peripheral samples. For instance, a study of SCZ patients and sex- and age-matched controls (age from 12 to 77 years) found that the incident of missing X chromosomes was found only in females. However, the frequency of X chromosome loss was significantly higher in female SCZ than in normal females (p<0.001) only in the 40- to 49-years-old age group. No significant differences of X chromosome loss were found in other age groups between SCZ and controls. One noteworthy thing is that the X chromosome alterations in SCZ had only been studied in the Brazilian population in this research, whether the phenomenon occurred in other people is unknown [43]. In addition, X chromosome abnormally seems significantly associated with age at onset of SCZ, particularly for the late onset. The frequency of the Turner’s syndrome (2/38,5.3%) and 47,XXX (1/38 ,2.6%) in childhood onset SCZ are higher than the frequency of the Turner’s syndrome (11/6483 ,0.17%)[29] and 47,XXX (22/8837 ,0.25%) [37] in adult onset SCZ, respectively [44]. In comparison with X chromosome aneuploidies, the abnormal passage of Y chromosome in SCZ is less reported, such as few case reports on males with XYY and XXYY. For example, one case of a 19-year-old white male with 47XYY karyotype was diagnosed SCZ by DSM-IV with aggressive behavior [45]. Another case found a 41-year-old caucasian man with 47XXYY karyotype who diagnosed in SCZ also showed impulsive aggression [46]. The reason for less Y chromosome aneuploidies reported in schizophrenia is related to the less commonality of extra copy of Y chromosome than the XXY or the XXX syndromes in general. In summary, it remains controversial as to whether sex chromosome abnormally, such as aneuploidies affect clinical symptoms of SCZ patients.

### Autism spectrum disorder (ASD)

ASD is a developmental disorder which is 4-5 times more common among boys than girls. While the biological mechanisms of the sex bias remain unclear, genetic studies showed that sex chromosomes genetic mutation is clearly associated with ASD. For example, mutation of X-linked genes, such as *NLGN3* and *NLGN4X*, have been associated with ASD phenotype as described previously [15–17]. It is also observed that kids with X chromosome abnormalities often have problems in social behavioral [47], social cognition [48] and increase levels of ASD traits [49], a typical symptom of ASD. For the X chromosome aneuploidies with extra copies, a study identified 14 ASD patients from total of 51 boys with Klinefelter syndrome (XXY) (27%) by ADI-R (the Autism Diagnostic Interview) [50], suggesting a possibility of extra copy of X chromosome may add more risk for ASD. Another study of children with an extra copy of X chromosome (*n* = 60), children with ASD (*n* = 58), or control kids (*n* = 106) found that kids (regardless boys or girls) with the extra copy of X chromosome expressed higher levels of social dysfunction and ASD symptoms than ASD and controls, particularly with increased social anxiety [47]. In addition, inactive X chromosome is also associated with ASD, such as Fragile X, a disease caused by a single mutated gene (FMR1 protein, FMRP) on X chromosome. For example, a study of 120 children (80 boys and 40 girls) with the fragile X and their unaffected siblings showed 30% of children with fragile X syndrome also has ASD [51]. Together, data suggest that both X chromosome aneuploidies and impairment of X chromosome activity can increase risk of ASD. Compared to X chromosome, the Y chromosome abnormalities are often less studied due to its complex and repetitive DNA sequence structure, which makes hard to detect using conventional genetic technologies. Nevertheless, several early studies showed that child carries XYY developed infantile ASD [52–54]. A study of 95 subjects with XXYY (age 1-55 years) demonstrated 92% language delay, 72% with ADHD, 28% with ASD, 47% with mood disorders in the individuals with XXYY [55]. A recent systematic review also showed the association between ASD and XYY syndrome and a description of two new cases with this association [56]. These reports suggested that additional genetic mutations in Y chromosome might be also involved in ASD.

### Attention deficit hyperactivity disorder (ADHD)

ADHD is other highly heterogeneous mental disorder. The incidence of ADHD in individuals with sex chromosome abnormality is higher than that in general population. There is a comprehensive study of sex chromosome aneuploidy in ADHD which included total of 167 participants with XXY (*n* = 56), XXX (*n* = 25), XYY (*n* = 33) and XXYY (*n* = 53), respectively. The results showed that 96 out of 167 (58%) individuals met DSM-IV criteria for ADHD, including 11 out of 56 (36%) individuals with XXY, 13 out of 25 (52%) in XXX, 25 out of 33 (76%) in XYY and 38 out of 53 (72%) in XXYY [57]. Another study reported an 18-fold increase in the prevalence of ADHD in girls with Turner’s Syndrome, and 4.8-fold increase in the risk of ADHD in combined boys and girls compared to the general population controls [58]. In addition to chromosome aneuploidies, a clinical case report found a 6 years old boy with ADHD carries a major de-novo Y chromosome abnormality, a deletion of the long arm and duplication of the short arm [59]. Together, while prevalence rates for ADHD in males and females is 2:1 in a general population, the sex differences in ADHD are increased significantly in the individuals carries sex chromosome aneuploidies as described [45].

### Mood disorders

Compare with other psychiatric disorder, the sex chromosome abnormality in mood disorders has been less investigated. It is known that females are more vulnerable to mood disorders in general. Although some case reports found the sex chromosome aneuploidies in BPD such as XXYY and XXX, none had evidence for aneuploidies increasing risk of BPD [60–62]. A study investigated the aneuploidies of sex chromosomes and risk of BPD in 1122 individuals with sex chromosome aneuploidy and showed a lower incidence of BPD in subjects with Turner’s syndrome and a higher BPD incidence in people carry 47, XYY. [41] The abnormalities of sex chromosome structure are also observed in a man with bipolar who has a deletion of the long arm of Y chromosome was identified in a karyotype analysis [63] and a man carries a ring Y chromosome [64]. Other studies of recurrent depressive disorder found higher frequency of Turner’s syndrome in depressive patients than that in regular population [65, 66]. While aneuploidy often expresses a notable phenotype caused by imbalance of gene dosage, one study of a family with two BPD patients (father and son) and one healthy member (mother) showed that the healthy mother carries more aneuploidic changes (51%) than the father (25%) and the son (7-11%) in the skin fibroblasts [67]. One related explanation for the unmatched phenotype with aneuploidies is the dynamic changes of x-linked escaping genes. A recent study of human lymphoblastoid cells showed that some X-linked escapee genes may be a common mechanism for development of psychiatric disorders between the patients with rare genetic diseases (XXY or XXX) and the general population of female with psychiatric disorders, including BPD [68].

## SEX CHROMOSOME INACTIVE HYPOTHESIS

While females (XX) carry twice as many X-linked genes on their sex chromosomes as males (XY), one X chromosome in females is active, and the other is inactivated in order to keep the sex chromosome dosage balance between males and females. The X chromosome inactivation (XCI) occurs randomly which is initiated by *Xist* gene [69] and regulated by *Tsix* gene [70] as well as *Ftx* gene [71] as a part of X chromosome inactive centre. However, XCI can also be non-random or skewed which is usually defined by at least 80% preferential inactivation of X chromosomes. The skewed XCI plays a role in the incidence and presentation of diseases in females, such as Rett syndrome, X-linked intellectual disability and X-linked adrenoleukodystrophy [72]. Recently, studies discovered a major role for XCI in generating female-specific, genetically directed stochastic diversity on spatial scales in mammals which might affect CNS function via regulating neuronal development within and between individuals [73]. It is hypothesized that the instability of XCI predisposes to psychosis [74] and autoimmune disease [75]. Although most of psychiatric diseases, such as ADHD, ASD, SCZ are closely related to early neuronal development, whether XCI abnormality is related to psychiatric is underinvestigated. One study found no major X-linked gene subject to XCI conferring susceptibility to 621 mothers who has an ASD children and 182 affected girls [76], while others found XCI skewness may be related with ASD by comparing 30 females ASD to 35 age- and sex-matched controls, and found higher frequency of increased XCI skewness in ASD [77]. There is one study investigated the XCI in mood disorders. They found that the genes for *Xist*, which initiation of XCI and escaping are significantly altered in the lymphoblastoid cells of female patients with BPD or MDD [68], suggesting an impairment of X chromosome function is involved in the mood disorders as showed in Table 2.

**Table 2:** Chromosomal rearrangements or anomalies associate with psychiatric disorder

Psychiatric diagnosis	Sex chromosome abnormality	Comments	study
SCZ	XYY	Case report, a 19-year-old white male with 47XYY karyotype diagnosed SCZ.	1998 [45]
	XXYY	Case report, a 41-year-old Caucasian man with 48XXYY karyotype who diagnosed in SCZ with impulsive aggression.	1996[46]
	45X	45X carriers showed 3 times more frequent in SCZ than general populations.	2000[38]
	45X/46XX	Case report, a 21-year-old female with TS diagnosed by DSM-IV.	2014[39]
	45X/46XX	Case report, a 41- year-old female with TS diagnosed by DSM-IV.	2010[40]
	45X/XX,47XXX	9/29 female SCZ showed X chromosome aneuploidy, chromosome X loss in the 40s year group was significantly higher in SCZ than in age-matched normal females.	2010[43]
	TS and 47XXX	TS (2/38) and 47XXX(1/38) in SCZ onset at childhood.	2008[44]
	46XX/47XXX/48XXXX	A SCZ female with 46XX/47XXX/48XXXX and 46XX/45Xkaryotype in her mother.	2010[35]
	XY/XXY, XYY, XO, XXX	XXY and XXX increase risk of SCZ. In 7519 SCZ patients, 41 carry XXY (0.54%), 21 carry XYY (0.28%). In 8837 SCZ, 56 carry XXX (0.63%).	1994[37]
ASD	XXY	14 of 51 XXY patients had diagnosis ASD by ASI-R.	2009[50]
	XXY	Case report, a16-year-old Caucasian male is diagnosed with ASD.	2007[47]
	XYY	A boy with 47XXY is diagnosed with ASD.	1998[53]
	XYY	A case report, a 7-year-old boy with XYY is diagnosed ASD.	2009[54]
	XYY	A case report, a four-year-old boy with 47XXY is diagnosed ASD.	1984[52]
	XYY	It is reviewed the publications found 42 ASD in 235 XYY, and reported two cases: a three-and-a-half-year-old male and two-and-a-half-year-old male both have ASD with 47XYY.	2014[56]
	XXYY	95 males with XXYY: ADHD (72.2%), autism spectrum disorders (28.3%), mood disorders (46.8%).	2008[55]
	XCI	No major X-linked locus is likely to be involved in these ASD families with skewed XCI in 621 mothers and 182 ASD girls.	2008[76]
	XCI	By analyzing 30 ASD females and 35 female controls, increased X chromosome skewness (e.g., >80:20%) was detected in autism group (33%) compared to controls (11%); but no mutation of *XIST* gene was found in both groups.	2005[77]
ADHD	XXY, XXX, XYY	Higher risk of ADHD: XYY males > XXY males > XXX females.	2012[57]
	XO	18-fold increase in the prevalence of ADHD in girls with TS (24%) compared with girls in the general population (1.3%) and a 4.8 fold increase when compared with boys and girls in the general population (5%).	2006[58]
	Fragment of Y	Case report, an 8-year-old male have ADHD with deletion of the long arm and duplication of the short arm in Y chromosome.	2008[59]
BPD	XXY	A case report, a BPD patient with Klinefelter's syndrome.	1997[64]
	XXYY	A case report, A man with 48XXYY syndrome is diagnosed BPD.	2005[65]
	XXYY, XXX	A review, it is reported 3 cases with 47XXX and 2 cases with 48XXYY.	1994[63]
	Missing Y	Case report, a 25-year-old male with a deletion of the long arm of Y chromosome is diagnosed BPD.	2005[66]
	Ring Y chromosome	Case report, a 31-year-old man carrying a ring Y chromosome is diagnosed BPD.	2007[68]
MDD	45X, 47XXY	It is found 45X and 47XXY by analyzing blood and Skin fibroblast samples from a BPD patient and his parents.	2012[60]
RDD	XO	The frequency of Turner’s syndrome in 2194 RDD females (0.14%) is higher than the frequency observed 17017 live-born infants (0.018%).	2016[61]
SCZ, BPD	XY/XXY, XYY, XO, XXX	1122 subjects with sex chromosome aneuploidy (45X n=313; 47XXX n=5; 47XXY n=641; 47XYY n=111). No subjects with 45X or 47XXX are SCZ or BPD, 13 XXY and 5 XYY are SCZ, 7 XXY and 2 XYY subjects are BPD.	2001[41]
MDD, BPD	XCI	*XIST* gene expression is higher in 36 female BPD compare to 38 MDD (14 males and 24 females) and 36 healthy female controls.	2015[67]

Notable: TS= Turner’s Syndrome, XCI= X Chromosome Inactive

## CONCLUSIONS

Sex differences in onset, clinical symptoms, prevalence as well as response to treatments in various psychiatric disorders have been well recognized. However, the underlying mechanisms remain unknown. In this review, we gathered the literatures and recent discoveries on sex chromosomal aberrations in SCZ, ASD, ADHD and major mood disorders, and highlighted the potential linkages between sex chromosome abnormalities and the psychiatric diseases. It offers a new insight to understand sex-specific pathogenesis of psychiatric disease. However, due to the limited numbers of studies on sex chromosome abnormalities in psychiatric disorders, there is still lacking of clear clue on the relationship between the sex chromosome and psychosis due to small sample size and inaccurate or insensitive methods of sex chromatin screening. In addition, there is a big gap between psychiatric patients with family history with mental disorder and sex chromosome aneuploidies. Therefore, future study full karyotyping of individuals with psychotic diseases, particularly with a strong family history of mental disorder is needed. Using the new powerful cytogenetic techniques, we may advance our search for sex specific aetiology of psychiatric illness
